# Efficacy, Safety and Anticancer Activity of Protein Nanoparticle-Based Delivery of Doxorubicin through Intravenous Administration in Rats

**DOI:** 10.1371/journal.pone.0051960

**Published:** 2012-12-21

**Authors:** Kishore Golla, Bhaskar Cherukuvada, Farhan Ahmed, Anand K. Kondapi

**Affiliations:** 1 Department of Biotechnology, University of Hyderabad, Hyderabad, India; 2 Department of Biochemistry, University of Hyderabad, Hyderabad, India; 3 Centre for Nanotechnology, University of Hyderabad, Hyderabad, India; Virginia Commonwealth University, United States of America

## Abstract

**Background and Aims:**

Doxorubicin is a potent anticancer drug and a major limiting factor that hinders therapeutic use as its high levels of systemic circulation often associated with various off-target effects, particularly cardiotoxicity. The present study focuses on evaluation of the efficacy of doxorubicin when it is loaded into the protein nanoparticles and delivered intravenously in rats bearing Hepatocellular carcinoma (HCC). The proteins selected as carrier were Apotransferrin and Lactoferrin, since the receptors for these two proteins are known to be over expressed on cancer cells due to their iron transport capacity.

**Methods:**

Doxorubicin loaded apotransferrin (Apodoxonano) and lactoferrin nanoparticles (Lactodoxonano) were prepared by sol-oil chemistry. HCC in the rats was induced by 100 mg/l of diethylnitrosamine (DENA) in drinking water for 8 weeks. Rats received 5 doses of 2 mg/kg drug equivalent nanoparticles through intravenous administration. Pharmacokinetics and toxicity of nanoformulations was evaluated in healthy rats and anticancer activity was studied in DENA treated rats. The anticancer activity was evaluated through counting of the liver nodules, H & E analysis and by estimating the expression levels of angiogenic and antitumor markers.

**Results:**

In rats treated with nanoformulations, the numbers of liver nodules were found to be significantly reduced. They showed highest drug accumulation in liver (22.4 and 19.5 µg/g). Both nanoformulations showed higher localization compared to doxorubicin (Doxo) when delivered in the absence of a carrier. Higher amounts of Doxo (195 µg/g) were removed through kidney, while Apodoxonano and Lactodoxonano showed only a minimal amount of removal (<40 µg/g), suggesting the extended bioavailability of Doxo when delivered through nanoformulation. Safety analysis shows minimal cardiotoxicity due to lower drug accumulation in heart in the case of nanoformulation.

**Conclusion:**

Drug delivery through nanoformulations not only minimizes the cardiotoxicity of doxorubicin but also enhances the efficacy and bioavailability of the drug in a target-specific manner.

## Introduction

Hepatocellular carcinoma (HCC) is increasing globally and represents a major health hazard worldwide leading to more than 5,98,000 deaths annually [1], [2].In the Asia-Pacific region, HCC incidence rate is almost similar to that of chronic hepatitis B (HBV) infection [3], while in Europe, North America and Australia it is clearly linked to Hepatitis C (HCV) infection [4]. In the initial stage of cancer development, a number of effective treatment modalities were employed which include liver resection, ablation and transplantation [5]. But in the intermediate and advanced stages, chemotherapy via different means is an indispensable treatment option that is available and its potential is limited due to poor prognosis and systemic toxicity. Despite the advances in surgery, radiation and chemotherapy, the prognosis for HCC still remains poor [6]. A number of chemotherapeutic agents are in use for HCC treatment which includes doxorubicin, cisplatin, taxanes, 5′Flurouracil etc., [7]–[10].

Chemotherapeutic agents can be administered through systemic circulation but patients who receive this treatment generally encounter serious life-threatening side effects viz., cardiac toxicity, myelosuppression, pain, nausea, vomiting, and alopecia [11]. To achieve an optimum localisation of drug into the tumour and to decrease systemic exposure, a number of efforts have been put forward including regional cancer therapy, trans arterial chemoembolization (TACE), which involves intermittent injection of a chemotherapeutic agent such as doxorubicin, mitomycin C, mixed with a viscous embolic material (e.g., lipiodol) [12]. Nanoparticles mediated delivery system is a promising approach for targeted drug delivery to HCC, since it offers sustained release of drug over a longer period of time, thus increasing the pharmacokinetic performance of the drug [13]. Nanoparticles functionalize with the receptor that gets over expressed on the surface of cancerous liver, thereby leading to preferential localization and hence an increase in the therapeutic index of the drug [14].

Transferrin receptors (Trf1) are shown to be over expressed in rat and human cancerous liver cells [15], [16] in order to meet high iron demand by highly active and rapidly proliferating cells. In the past, successful attempts have been made to couple transferrin protein to different nanoparticles for targeted delivery to cancerous cells [14]. Compared to free administration of free doxorubicin, doxorubicin-loaded Apotransferrin nanoparticles have been shown to deliver the drug more effectively with significant activity against cell-mediated ascitic liver cancer upon localized administration through intra peritoneal route [17]. Liver cells uptake transferrin-bound iron through receptor mediated endocytosis into a low-density vesicle compartment of hepatocytes followed by the release of iron and recycling of transferrin [18]. In addition to transferrin receptor 1 [19], trans-membrane protein divalent metal transporter 1 (DMT1) [20], divalent metal transporter ZIP14 [21] were also reported to be involved in transferrin mediated iron transport. In the present study, we have employed doxorubicin-loaded nanoformulations of transferrin family of proteins, Apotransferrin (Apodoxonano) and Lactoferrin (Lactodoxonano). Efficacy was evaluated in Diethyl nitrosamine (DENA) induced HCC in Wistar rats. We have demonstrated the advantage of drug delivery in terms of improved efficacy, safety and pharmacological profile of nanoformulated doxorubicin compared to those with free administration of doxorubicin (Doxo), when administered through intravenous route.

## Materials and Methods

### Materials

Doxorubicin was a pharmaceutical preparation of Biochem Pharmaceutical Industries, Pune, India. Lactoferrin was purified from milk.

### Purification of Apotransferrin

Apotransferrin was purified from human blood by following the method of Cohn et al [22]. Human blood was obtained from the healthy volunteers for purification of Apotransferrin was obtained through written informed consent as per approval of the Institutional Ethics committee, University of Hyderabad.

### Purification of Lactoferrin from milk

The purification was carried out as per the method of Sharma et al., [23]. Briefly, the cow milk was defatted by centrifugation at 8000 rpm/10 min/4°C and diluted with 0.05 M Tris–HCl (pH 8.0). CM-Sephadex was added to it (7 g/l) and stirred slowly by a mechanical stirrer for an hour. The gel was allowed to settle and the milk was decanted. The gel was washed with excess of 0.05 M Tris–HCl and packed into a column. Washing was carried out with buffer containing 0.1 M NaCl followed by elution of lactoferrin with the same buffer containing 0.25 M NaCl. The protein was passed through a Sephadex G-100 column (2×100 cm) in 0.05 M Tris–HCl (pH 8.0). The purity was confirmed on SDS–PAGE. All other reagents, biochemical analysis kits and biochemicals were of analytical and molecular biological grade.

### Preparation and characterization of nanoparticles

Doxorubicin loaded apotransferrin nanoparticles (Apodoxonano): Apotransferrin nanoparticles were prepared by sol-oil chemistry protocol described [Indian patent # 1572/CHE/2006; 17]. Doxorubicin loaded lactoferrin nanoparticles (Lactodoxonano): Lactoferrin nanoparticles were prepared based onthe method of Krishna et al., 2009 [17] with some modifications [Indian patent # 4657/CHE/2011 dated 30.12.2011]. The particle formation of lactoferrin-doxorubicin hydrochloride (doxo) in oil phase was initiated by sonication using power ranges from 50–80% and probe used was 0.375 inches of diameter solid titanium tip (Cat no 0-120-0009) on ultrasonic homogenizer (Model 300V/T of Bioloics Inc., USA) and by passing 2 seconds pulses with a gap of 2 seconds between successive pulses over a period of 15 min. After sonication of the olive oil containing lactoferrin-doxorubicin hydrochloride, the resulting mixture was immediately frozen in liquid nitrogen at −196°C for 10 min. Then it was kept on ice for 4 hours. The Particles formed were separated by centrifugation at 6000 rpm for 10 minutes. After the supernatant was decanted, the pellet containing the nanoparticles of lactoferrin and doxorubicin hydrochloride was washed twice using 15 ml of ice cold diethyl ether. The pellets were immediately dispersed thoroughly by manual vortexing in 1 ml of phosphate buffered saline and used for experiments. Particles were characterized using scanning electron microscope (SEM), transmission electron microscope (TEM) and atomic force microscope (AFM) as per manufacturer's manual.

### Hemolysis test

The erythrocyte toxicity assay was conducted by using the protocol of Zhang et al (2007) [24]. In brief, 100 µl of the erythrocyte stock dispersion was added to 1 ml (1 mg/ml and 2 mg/ml) of Doxo, Lactodoxonano and Apodoxonano suspension. Incubation was carried out at 37°C for different periods of time. After incubation under shaking, debris and intact erythrocytes were removed by centrifugation at 1200 rpm for 5 minutes. Then, 100 µl of the resulting supernatant was dissolved in 2 ml of an ethanol-HCl mixture (39∶1; 99% ethanol [v/v], 37% hydrochloric acid [w/v]). This mixture dissolved all the components but could not prevent precipitation of hemoglobin. A saline solution was used as a negative control (0% lysis), and double distilled water was used as a positive control (100% lysis). The absorbance of the supernatant was determined at 398 nm by UV- Visible spectrophotometer (Jasco V550).

The hemolysis rate (HR %) was calculated using following equation:
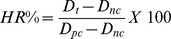
Where D_t_, D_nc_, and D_pc_ are the absorbance of the tested sample, the negative control and the positive control, respectively. The experiments were run in triplicate.

### Animal Study design

Male Wistar rats (4–6 months old; Weight: 0.155–0.175 kg)) were housed in cages and kept on a 12 h light/dark cycle. Food and water were given ad libitum. Following acclimatization, rats were randomly divided into seven groups (6 animals per group at 15 min, 30 min, 1, 4, 8, 12 and 24 hr). All drugs were delivered intravenously through tail vein. All studies were conducted in accordance with the animal ethics guidelines for the care and use of laboratory animals, which were housed in a facility accredited by the animal ethics committee, University of Hyderabad. Animals received either 3.2 mg/kg single dose of doxorubicin or an equivalent dose of doxorubicin contained in the protein nanoparticles mentioned. After the completion of time points, blood samples were collected by heart puncture before sacrificing the rats after 15 min, 30 min, 1, 4, 8, 16 and 24 h time points. Then, animals were sacrificed by cervical dislocation and tissues viz., brain, heart, lungs, spleen, liver, kidneys and bone marrow were isolated. Tissues were homogenized in PBS with homogenizer. From1 ml of the homogenized tissue extracts, protein was precipitated by adding 300 µl of 30% silver nitrate in double distilled water. The contents were mixed in 3 ml of methanol and vortexed for extraction of the drug into methanol. Extracts were centrifuged at 5000 rpm for 10 min at 4°C. Doxorubicin was separated and estimated using spectrophotometer at 470 nm [17]. Blood and tissues were processed for tissue distribution and pharmacokinetic analysis at the above time points.

### Pharmacokinetics

Pharmacokinetic parameters were computed using AUC steady state kinetics with Lz method of Kinetica v5.0 software (Thermo Scientific).

### Anticancer activity of Apodoxonano, Lactodoxonano and Doxo in DENA treated rats

#### Induction of HCC [25]

Male Wistar rats (4–6 months old; Weight: 0.155–0.175 kg) were housed in cages and kept on a 12 h light/dark cycle. Following the acclimatization for one week, HCC was induced in rats by adding 100 µg/l of DENA in drinking water for 8 weeks.

#### Treatment

Anticancer experiments were carried out using 5 rats (cancer-induced as above) in each group. The rats were randomly divided into 4 groups and maintained for one week. First group consists of cancer-induced (by DENA treatment) rats without any drug treatment. Second, third and fourth group corresponds to the cancer-induced rats treated with Doxo, Apodoxonano and Lactodoxonano respectively. Drug treatment was started at the end of one week after cancer induction and dose of 2 mg/kg of Doxo or Doxo equivalent of indicated protein nanoparticles were administered through intravenous route under sodiumpentabarbitrate anesthesia in the tail vein on 1, 7, 14, 21 and 28 day.

#### Analysis

During treatment, weight of animals was monitored. Following treatment completion on day 7, blood was drawn by heart puncture and animals were sacrificed under sodium pentabarbitrate anesthesia. The liver was removed and lobes were separated. Tumor nodules on the surface of each lobe measuring>3 mm in diameter (measured with a digital caliper) were counted and the difference in the number of nodules in the four experimental groups was statistically evaluated. A second evaluation of tumor growth was performed after the lobes were fixed in 10% formalin, which makes neoplastic nodules more evident. The upper and lower surfaces of each fixed lobe, together with a millimeter graded bar were photographed for the digital enlargements of the photos. Since lumps smaller than 2 mm could not be identified with much certainty as nodules, these were not counted. In livers displaying a multi-nodular surface, identifiable nodules were counted to a maximum of 50, since the total number could not be reliably assessed. The antitumor effect of the drugs was estimated by comparing the number of animals with more than 50 tumor masses in each of the different experimental groups. Samples of all the lobes of the liver were fixed in 10% formalin embedded in paraffin and were routinely stained with hematoxylin and eosin (H&E).

Tissue sections were prepared from liver, heart, spleen, kidneys followed by the homogenization of the remaining tissue in PBS for estimating the Doxo in them as per the protocol described previously.

#### Safety Analysis

Safety analysis was done by using biochemical kits manufactured by Qualigens, India for Serum glutamic oxaloacetic transaminase (SGOT), Serum glutamic pyruvic transaminase (SGPT), Creatinine and Blood Urea Nitrogen.

#### Lactate Dehydrogenase Assay [26]

The reaction velocity is determined by a decrease in absorbance at 340 nm resulting from the oxidation of NADH. One unit causes the oxidation of one micromole of NADH per minute at 25°C and pH 7.3, under the specified conditions. Incubation of the spectrophotometer was done for 4–5 minutes to achieve temperature equilibration and blank rate was established. 300 µl of serum was added to 2.5 ml of 0.2 M Tris⋅HCl (pH 7.3), 0.1 ml of 6.6 mM NADH and 0.1 ml of 30 mM Sodium pyruvate to record ΔA_340_/min from initial linear portion.

#### Glutathione peroxidase (GPX) Assay [27]

The extent of oxidation of NADPH+H^+^ is taken as the measure of the activity of GPX. The reaction mixture contained 660 µl of phosphate buffer, 1 U of GR i.e. 20 µl, 100 µl of GSH solution, 100 µl of NADPH solution and 10 µl of heart tissue homogenate. Phosphate buffer alone acts as blank. The mixture was incubated for 5 min to allow H_2_O_2_ free oxidation of NADPH and to obtain a base line at 340 nm. The reaction was started by adding 100 µl of H_2_O_2_ and change in the absorbance at 340 nm was monitored for 5 min at one min intervals.

#### RNA isolation, reverse transcription and real-time polymerase chain reaction [28]

Total RNA was prepared from the frozen liver tissue using Trisol Reagent (Invitrogen, USA). Reverse transcription and semi-quantitative polymerase chain reaction analysis was carried out as per the manufacturer's protocol (Superscript III First strand synthesis kit, Invitrogen); data was normalized to the expression of the GAPDH which is used as internal control. The following primers were used: p53: sense CCATGAGCGTTGCTCTGATG, antisense TTATCCGGGTGGAAGGAAATC; p21: sense CCTGTTCCACACAGGAGCAA, antisense GATTGCGATGCGCTCATG; VEGFR1; sense: CGTACCCGCAACGGAGAA, antisense: GCGTCCTCGGCAGTTACATC. Liver of healthy rat was used as control.

#### Estimation of TNF alpha in serum

TNF- α was estimated in the serum by Rat TNF-alpha Platinum ELISA kit (Bender MedSystems, BMS622) as per the procedure supplied with the kit. Briefly, after washing the microwell strips with wash buffer, 50 µl of serum was added in duplicate along with 50 µl of sample diluent to the sample wells, whereas 100 µl of sample diluent was added in duplicate to the blank wells. Following incubation for 2 hours at room temperature, wells were washed 4 times and 50 µl of Biotin-Conjugate was added to all wells and covered with an adhesive film and incubated at room temperature for 2 hours. After washing the microwell strips for 4 times, 100 µl of diluted Streptavidin-HRP was added to all wells and incubated at room temperature for 1 hour. The microwell strips were washed for 4 times and 100 µl of TMB substrate solution was added to each well followed by incubation for 10 min. Substrate development is stopped by addition of 100 µl stop solution and absorption was measured at 450 nm in a micro well plate reader.

#### Data analysis

Concentrations of doxorubicin at each time point in liver, kidney, spleen, brain, heart, lungs, bone marrow and plasma of the rats for both soluble and nanoparticle formulations were randomly assigned to create concentration/time profiles. Pharmacokinetic parameters were determined by noncompartmental analysis using Kinetica v5.0, (Thermo Fisher Informatics, USA) with a single-dose *i.v.* bolus drug administration model. The mean weights in the young rat groups were used for normalization of pharmacokinetic parameters. The data was analyzed by one-way ANOVA with age and treatment as factors using Sigma Stat. The level of statistical significance was P<0.05. Data are expressed as means±SD.

## Results

**Figure 1:**
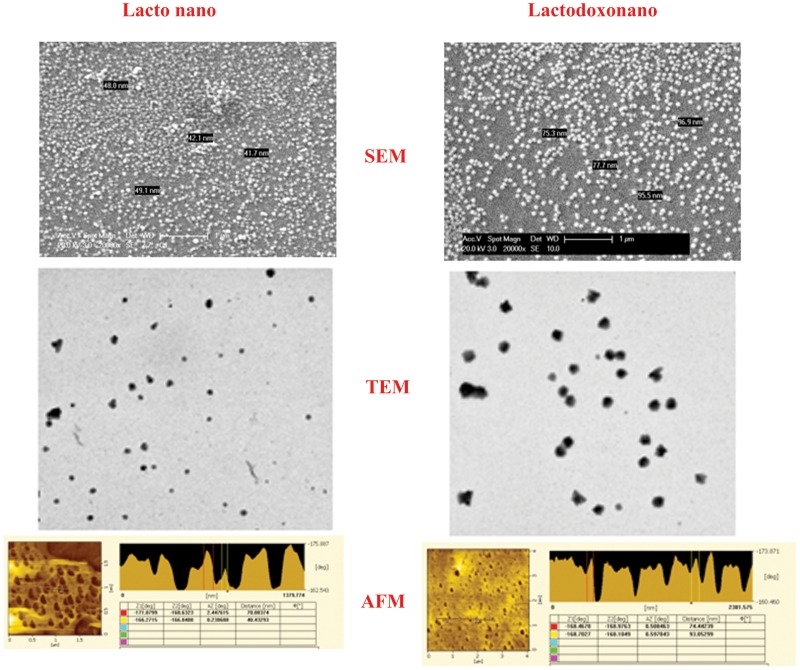
Characterization of lactoferrin nanoparticle size by SEM, TEM and AFM.

Doxorubicin loaded with Apotransferrin and Lactoferrin nanoparticles are spherical and dispersed uniformly with an average diameter of around 70 to 80 (17) nm and 68 nm (Fig. 1) respectively. Estimation of doxorubicin showed 66% of drug encapsulation in Apodoxonano, while 79% in Lactodoxonano, suggesting the higher drug loading capacity of Lactodoxonano (Table S1). Analysis of drug retention in protein nanoparticles showed that the nanoparticles are quite stable with only 2.5% drug loss when stored until 24 hours and only 5% loss when stored in suspension for 3 months (Table S2).

### Hemolytic analysis of nanoformulations in the absence of doxorubicin

Hemolysis of the blood is an important problem associated with the bioincompatibility of materials. Erythrocytes get hemolysed upon contact with deionized water. This problem may be aggravated in the presence of an implantable material. Less than 5% hemolysis is generally regarded as a nontoxic effect level. Hemolysis rates of fresh human blood with apotransferrin and lactoferrin nanoparticles observed in the present study is based on protocol used by Rao and Sharma (36) and is shown in Table 1. Increase in the apotransferrin and lactoferrin nanoparticle concentration and incubation time caused only an insignificant change in the hemolytic activity. However, HR % of apotransferrin and lactoferrin nanoparticles at two concentrations for different time periods was observed to be below 2%. No broken erythrocytes were visible under microscopic examination and the morphology of cells remained normal, indicating the low erythrocyte membrane-damaging effect of apotransferrin and lactoferrin nanoparticles (Table 1).

**Table 1:** Hemolysis test.

	1 mg/ml		2 mg/ml	
Incubation Time	30 min	60 min	30 min	60 min
**Apodoxonano**	0.47	0.61	0.91	1.23
**Lactodoxonano**	0.41	0.72	1.01	1.32
**Doxorubicin**	3.4	4.7	4.2	5.7

Positive control: 100% .

Negative control: 0.002% .

### Tissue distribution and Pharmacokinetics of nanoformulations of doxorubicin

**Figure 2:**
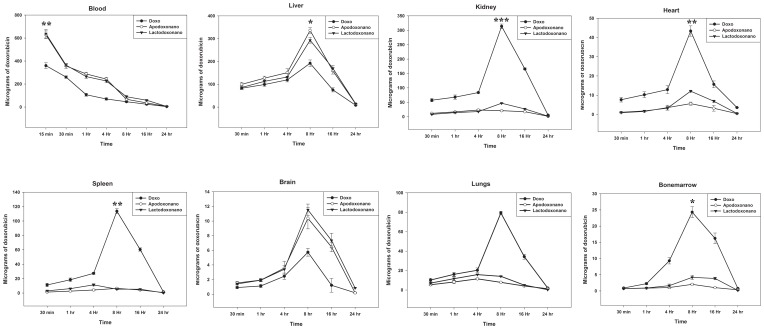
Intravenous administration of Lactodoxonano, Apodoxonano and Doxo. Lactodoxonano, Apodoxonano and oxo (800 µg of drug equivalent) were administered intravenously (*i.v.*) and after indicated time points animals were sacrificed. Brain, Liver, Heart, Kidney, Spleen, Lungs, Bone marrow and Blood were collected. Proteins were precipitated in 30% AgNO_3_ and drug was extracted in methanol. Drug was separated and estimated using absorption at 480 nm.

Doxorubicin levels in plasma along with different organs were determined following a single injection of nanoparticles loaded with doxorubicin (3.2 mg/kg Doxo equivalence) in Wistar rats. The levels observed over a 24 hr time period in those tissues are shown in Fig. 2 and the pharmacokinetic parameters were determined by Kinetica v 5.0 program are presented in (Table 2). The concentration of Doxorubicin in the blood after 4 hr was found to be above 200 µg when injected with both nanoformulations and around 69 µg in the absence of any carrier molecule. This explains the relative stability of doxorubicin in nanoformulation against plasma clearance. Further this does not lead to any non-specific accumulation of the drug in various organs leading to toxicity which in contrast is a characteristic feature of doxorubicin without a delivery vehicle. At this time point only liver has shown the highest accumulation of drug amounting to >100 µg/ml. This is followed by kidney, spleen and lungs which have shown accumulations above 50 µg/ml, 20 µg/ml and<20 µg/ml respectively. Less than 5 µg/ml of the drug was present in the remaining organs viz., Heart, Brain and Bone marrow. The doxorubicin administered without any carrier vehicle showed a steep 2–4 fold increase in its level in heart, kidney, spleen, bone marrow and lungs at 8 hr post injection. On the other hand there was a concomitant decrease of doxorubicin level in plasma and an increase in liver when injected with either of nanoformulations i.e., Apodoxonano and Lactodoxonano.

**Table 2:** Pharmacokinetics profile of Lactodoxonano, Apodoxonano and Doxo.

		AUC	AUMC	C_max_	T_max_	T_1/2α_	T_1/2_
		(h)*(µg)	(h)∧2*(µg)	µg	Hr	Min	Hr
**Plasma**	**Doxo**	1135 (56.8/ml)	7166 (358/ml)	360 (18/ml)	0.25	<15	2.8*
	**Apodoxonano**	2305 (115/ml)	12851 (642/ml)	627 (31.39/ml)	0.25	28	2.9*
	**Lactodoxonano**	2514 (125/ml)	15836 (791/ml)	637 (31.88/ml)	0.25	28	2.2*
**Kidney**	**Doxo**	3317 (2073/g)	34815 (21759/g)	313 (196/g)	8	---	1.6
	**Apodoxonano**	366 (229/g)	3524 (2202/g)	23.1 (14.4/g)	4	---	2.1
	**Lactodoxonano**	553 (345/g)	5878 (3674/g)	46.8 (29/g)	8	---	2.1
**Spleen**	**Doxo**	1172	12506	113	8	---	1.55
	**Apodoxonano**	95	1056	6.43	8	---	3.1
	**Lactodoxonano**	118	1032	11.38	4	---	1.8
**Liver**	**Doxo**	2269 (153/g)	21400 (1426/g)	192 (12.8/g)	8	---	2.42
	**Apodoxonano**	3897 (259/g)	39311 (2620/g)	336 (22.4)	8	---	2.23
	**Lactodoxonano**	3665 (244/g)	38353 (2556/g)	293 (19.4)	8	---	2.24
	**Doxo**	457	5173	43.4	8	---	3.8
**Heart**	**Apodoxonano**	73	787	5.6	8	---	2.7
	**Lactodoxonano**	134	1500	12.1	8	---	2.1

Values in the parenthesis indicates the concentration of doxorubicin in micrograms per ml of blood/per gram of tissue;

* value of t_1/2β._

**Pharmacokinetic parameters**.

**AUC:** The integral of the concentration-time curve (after a single dose or in steady state).

**AUMC:** Partial area under the moment curve between t start and t end.

**t ½:** The time required for the concentration of the drug to reach half of its original value. In serum, t_1/2α_ is referred duirngdistribution phase and t_1/2β_ during elimination phases.

**C_max_ :**The peak plasma concentration of a drug after IV administration.

**T_max_ :**Time to reach C_max._

### Safety analysis

**Figure 3:**
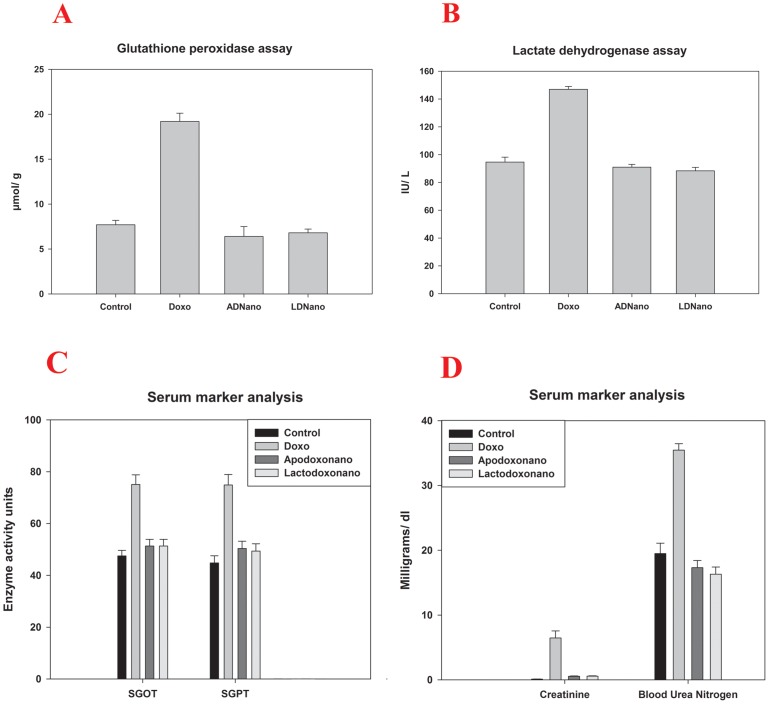
Safety analysis in healthy rats. Lactodoxonano, Apodoxonano and Doxo (800 µg of drug equivalent) were administered intravenously (*i.v.*). Safety analysis was carried out using biochemical kits. Heart toxicity was measured in terms of Glutathione peroxidase and LDH. Both the enzymes showed minimal expression when treated with nanoformulations as that of the control but gets increased during Doxo treatment. Kidney toxicity was evaluated by the levels of Creatinine, Blood Urea Nitrogen, whereas the Liver toxicity was estimated in terms of SGOT and SGPT levels. The level of these 4 markers also followed the same pattern as that of Glutathione peroxidase and LDH i.e., they showed minimal levels as that of control when treated with nanoformulations opposite to Doxo.

**Figure 4:**
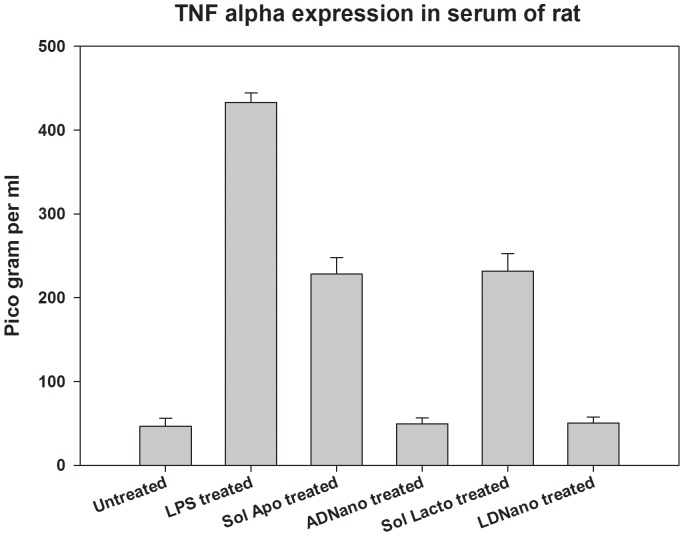
TNF-α expression. TNF- α in 50 µl of serum was estimated using Rat TNF-alpha platinum ELISA kit (Bender MedSystems) as per the procedure supplied with the kit. LPS was used as positive control. The immunogenicity of lactoferrin and apotransferrin is evident by the increased expression of TNF-α, a proinflammatory cytokine when treated with soluble forms of both proteins. The results showed maximum expression of TNF-α in LPS treatment (positive control). But when administered with Lactdoxonano (LD nano) and Apodoxonano (AD nano),TNF-α level is minimal as that of untreated control suggesting negligible inflammation caused by the nanoformulations.

A major concern in the doxorubicin treatment is the cardiotoxicity, which can be estimated by Glutathione Peroxidase activity in heart tissue homogenate and observation of LDH levels in serum. We have therefore estimated Glutathione Peroxidase in heart tissue homogenate of the treated rat and LDH levels in serum. The results presented in Fig. 3 (Panel A) clearly show that the Glutathione Peroxidase activity is significantly increased in heart tissue homogenate of the rat treated with doxo, while its levels in the animals treated with nanoformulations remain identical with that of control untreated animal. To further confirm this, we have measured LDH levels in serum and the results show that it is present in serum at detectable levels in doxo treated animals (Fig. 3 Panel B) and is absent in the serum of animals treated with nanoformulations. This suggests that doxo indeed caused damage to heart, which is prevented when treated with nanoformulations. Further, we have analysed the liver and kidney toxicity of the doxo versus nanoformulations by monitoring the levels of SGOT, SGPT, creatinine and blood urea. The results show that toxicity of doxo is high in liver and kidney, which however gets drastically reduced when administered through nanoparticles (Fig. 3 Panel C and D). Immunogenecity of the nanoformulations was tested by checking the TNF-α level in the serum. Here the soluble forms of both proteins exhibited significant expression of TNF-α whereas the nanoformulations did not when compared to LPS which is taken as positive control (Fig. 4). The results thus demonstrate that the delivery of doxorubicin through nanoparticles is much safer and efficient.

### Treatment of Hepatocellular Carcinoma

#### Histological analysis of Treated rats

**Figure 5:**
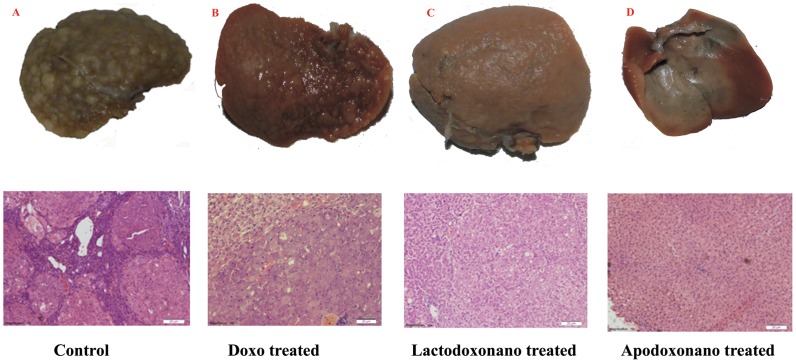
Treatment of hepatocellular carcinoma. Hepatocellular carcinoma was induced by adding 100 mg/L of DENA in drinking water for eight weeks. After one week of gap from the DENA treatment, animals received 2 µg/g of drug equivalent of Apodoxonano, Lactodoxonano and Doxo through *intra venous* administration on 1, 7, 14, 21 and 28 day. Panel Ashows untreated liver with maximum number of nodules which were reduced to some extent when treated with doxo (panel B). HCC was significantly reduced when treated with Lactodoxonano (Panel C) and Apodoxonano (Panel D) , which is indicated by the reduction in the number of nodules. It is further analyzed by H & E analysis of liver nodules along with treatments.

**Figure 6:**
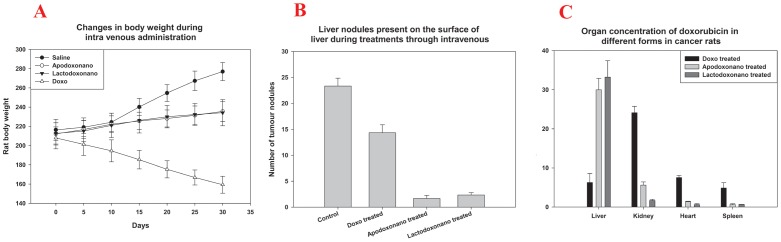
Efficacy against hepatocellular carcinoma. Panel -A shows changes of rat body weight during treatments. The body weights of rats were decreased drastically when treated with Doxo and increased when given saline. During the treatment with nanoformulations , a slight increase in body weight was observed. Panel-B shows tumour nodules on the surface of liver. Tumour nodules are reduced significantly when treated with Apodoxonano and Lactodoxonano, when compared with Doxo and untreated rat (control). Panel C: After treatment of hepatocellular carcinoma, animals were sacrificed to collect the organs for their subsequent estimation of Doxo levels. Here the highest levels of drug were found only in the liver of rats treated with nanoformulations due to its specificity when compared to Doxo.

HCC is induced by oral administration of DENA. These rats showed significant HCC formation as evidenced by the formation of nodules on liver tissue and also from the cancerous cellular growth profile in liver histochemical sections (Fig. 5A; 6B). When these rats were treated with Doxo, the nodule formation was found relatively reduced with some cancerous growth still evident in liver sections (Fig. 5B; 6B). But when the animals were treated with Apodoxonano and Lactodoxonano, most of the nodules have disappeared suggesting maximum inhibition of HCC, which is further confirmed by histochemical analysis, where the liver tissue sections showed normal cellular morphology (Fig. 5 C and D and 6B). These observations suggest that the protein nanoparticle formulations of apotransferrin and lactoferrin are highly efficacious compared to soluble form of doxorubicin.

#### Mean body weight

DENA induced HCC rat shows an increase in mean body weight (MBW).When treated with Doxo, its MBW is decreased significantly indicating the severe toxic effects exerted by the drug (Fig. 6A),while in the case of treatment with Apodoxonano and Lactodoxonano the MBW of animals was found significantly restored (Fig. 6A) with a slight decrease in weight due to inhibition of cancer growth. Thus nanoparticle encapsulation of Doxo has reduced the Doxo-related weight loss establishing its reduced off-target effects when delivered with protein nanoparticles.

#### Doxorubicin concentration in different organs of DENA treated rats

The levels of doxorubicin in the tissue homogenate of organs liver, kidney, heart, spleen have been analyzed to assess its accumulation during the treatment. The results presented in Fig. 6C show that highest levels of doxorubicin are present in liver, while the levels were significantly lower in kidney, heart and spleen when administered through Apodoxonano and Lactodoxonano compared to Doxo. Since the liver is the site of action as well as the site of cancer induction, the presence of higher drug levels when administered through Apodoxonano and Lactodoxonano would help in enhancing the efficacy of treatment.

#### Analysis of progression of HCC through biomarker analysis

**Figure 7:**
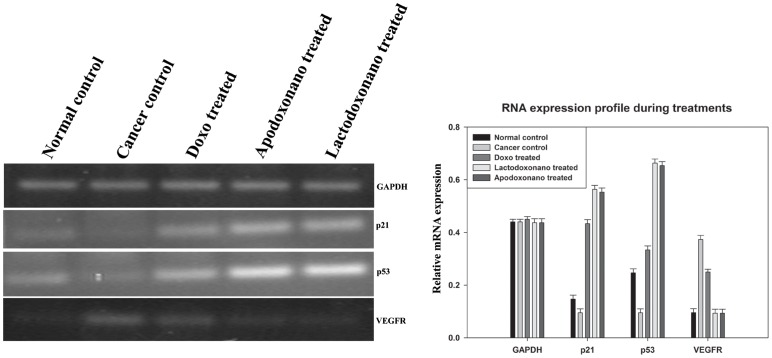
Molecular marker analysis. Analysis using molecular markers showed that **t**umour reduction was better during Lactodoxonano and Apodoxonano treatment when compared to Doxo, which is indicated by the over expression of the major tumoursuppressor genes namely p53 and p21. On the other hand, reduced vasculature due to decreased levels of VEGFR during nanoformulation treatment also reinforces its efficacy.

The major proteins that play a vital role in controlling the cancer namely, p53 and p21 which act in the same pathway undergo upregulation during the regression of cancer under tumor suppressing conditions. Accordingly, the levels of p53 and p21 were analyzed in treated rats and the results presented in Fig. 7 show that these levels were significantly up-regulated when treated with doxorubicin, while the rats treated with Apodoxonano and Lactodoxonano showed highest upregulation. Furthermore Doxo treatment shows comparatively lower enhancement of p53 compared to p21 (Fig. 7). Additionally, the analysis of vascular development in cancer tissue is analyzed by monitoring VEGFR levels. The results show that VEGFR which is elevated during cancer prognosis is significantly inhibited in Apodoxonano and Lactodoxonano treated rats, while Doxo treated rats showed a relatively lower decrease of VEGFR expression (Fig. 6). These results together point out that Apodoxonano and Lactodoxonano enhance tumor suppressing environment and block vascular development effectively through targeted localization of drug in cancer tissue.

## Discussion

To overcome toxicities caused by anthracyclins like doxorubicin in cancer treatment, various formulations were developed using nanoparticles made up of different biodegradable polymers [29]. They include microspheres [30], liposomes [31]–[33], polymerosomes [34]–[37], polymeric micelles [38]–[40] and stealth and non-stealth solid lipid nanoparticles [41], [42] made up of proteins, dextrans, acrylates, urethanes, PEG etc.. Out of these different formulations, PEG is used widely as block copolymer for the preparation of nanoparticles since they escape from the clearance induced by reticuloendothelial system (RES) efficiently, which is rather less pronounced in the case of many colloidal carriers. The present protein nanoformulations of doxorubicin were turned out to be more efficient with better pharmacokinetic performance than the existing vehicles. In any cancer treatment, the main emphasis is to improve the tumor- plasma ratio and reduce the off-target effects. Even though the local chemotherapy shows promising results with minimal toxicity [43] there is need to design not only a novel delivery system for administration but also in getting the technical expertise to identify the exact tumor location followed by appropriate treatment modalities. The present study addresses the above issues by exploiting the over expression of apotransferrin and lactoferrin receptors on cancer cells [44], [45]. The nanoparticles of apotransferrin and lactoferrin proteins will serve as potential ligands for delivery of doxorubicin. Since the physically encapsulated doxorubicin has a more pronounced effect than the covalently bound form [46], we prepared the nanoparticles of doxorubicin with the above proteins without any chemical coupling of the drug with the proteins (Patents applications #1572/CHE/2006; #4657/CHE/2011). One of the important features of the present formulations is the amount of doxorubicin used in cancer treatment (2 mg/kg body weight), which iswell below the usually allowed dosage that ranges from 7.5 to 12.5 mg/kg in other studies [47]–[49].

We compared plasma concentrations of Doxo after a single intravenous injection of nanoparticles equivalent to 3.2 mg/kg bodyweight of doxo. Since the amount injected is relatively lower, the distribution of doxorubicin in all the tissues was determined at seven time points: 15 min, 30 min, 1, 4, 8, 16, 24 hr post injection. Even though the plasma C_max_ was found to be increased by more than 50% for both the nanoformulations, there was no apparent change in T_max_. This was also reflected in the AUC, which is found much higher for the apotransferrin and lactoferrin formulations compared to Doxo. The plasma AUC profiles of these nanoformulations are standard biexponential. AUC gets enhanced in the case of blood(>100%) and liver (>50%) when administered with nanoformulations but gets drastically reduced by 4–10 fold in all the remaining tissues. This specific and enhanced accumulation in liver i.e., at the site of the tumor, when compared to the other tissues is due to the selectivity of the nanoformulations used. Apodoxonano as well as Lactodoxonano showed similar pharmacokinetic (PK) profile with two fold higher AUC and t_1/2α_ compared to Doxo, while no change in t_1/2β_. Higher t_1/2α_exhibited by Apodoxonano and Lactodoxonano indicates an increase of drug in blood circulation as also seen from an increase in AUC_0-∞_. Indeed, similar PK enhancement from Doxo was observed in case doxorubicin loaded poly (alkylcyanoacrylate) nanoparticles (PACA-NPs) produced by redox radical emulsion polymerization (RREP) [50] in terms of AUC_0-∞_, t_1/2α_ and t_1/2β_. When solid lipid nanospheres are employed [51], the AUC_0-∞_ enhancement compared to Doxo was 17.47 fold with no change in t_1/2α_ and t_1/2β_. While in the case of doxorubicin-loaded (PEG)_3_-PLA nanopolymersomes [52], theAUC_0-∞_ enhancement compared to Doxo was 149-fold, but the T_1/2_ was 44.69 suggesting higher resident time and longer bioavailability. Indeed, this is the feature of the liposomal formulations where they exhibit higher AUC_0-∞_ (Li et al., 2009) [53].

The more specific accumulation of the drug in the liver relative to the other tissues at 8 hr post injection may be attributed to the specific interaction of the nanoparticles with the corresponding receptors that gets over expressed. Cardiotoxicity and renal toxicity which were the major limiting factors of the doxorubicin treatment were significantly reduced with these new formulations which was reflected not only in the levels of drug accumulation in the organs but also in the expression of markers like Glutathione peroxidase and LDH etc.

The apparent absence of bodyweight reduction and the insignificant hemolysis in the animals treated with nanoformulations further support the efficacy of these novel formulations with negligible toxicity. The treatment efficacy is further indicated by the reduction of tumor nodules and the expression of the two tumor suppressor proteins, p53 and p21 and the concomitant repression of VEGFR levels. Finally the improved profile of nanoformulations used here provides an opportunity to increase the amount of drug which can be targeted to the tumor directly with minimum toxicities. This in turn will help in the development of drug regimens with higher doses while maintaining increased efficacy levels intact with less pronounced side effects.

In conclusion, nanoparticle formulation of apotransferrin and lactoferrin are efficacious and safe with the enhanced bioavailability of drug in target tissue and plasma.

## Supporting Information

Table S1 **The encapsulation efficiency of nanoformulations was measured in nanopartciles prepared with 10 mg of protein and 10 mg of drug.** When drug and protein was estimated in nanopartciles, the results showed that Apotransferrin and Lactoferrin encapsulated 66% and 79% respectively.
(DOCX)

Table S2 **The stability of nanoparticles was monitored at 4°C by incubation over 90 days.** The results shows that both the formulations exhibited 2.5–5% drug loss when incubation period was between 24 hr to 90 days.
(DOCX)

## References

[1] Bosch FX, Ribes J, Diaz M, Cleries R (2004) Primary liver cancer worldwide incidence and trends. Gastroenterology 127: S5–S16. 15508102 10.1053/j.gastro.2004.09.011

[2] Parkin DM, Bray F, Ferlay J, Pisani P (2002) Global cancer statistics, 2002, CA CancerJ. Clin 55 2: 74–108. 10.3322/canjclin.55.2.74 15761078

[3] Yang HI, Lu SN, Liaw YF, You SL, Sun CA, et al (2002) Hepatitis B e antigen and the risk of hepatocellular carcinoma. NEJM 347 3: 168–74. 12124405 10.1056/NEJMoa013215

[4] Colombo M, Kuo G, Choo QL, Donato MF, Del Ninno E, et al (1989) Prevalence of antibodies to hepatitis C virus in Italian patients with hepatocellular carcinoma . Lancet 2 8670: 1006–1008. 2572740 10.1016/s0140-6736(89)91016-7

[5] Varela M, Real MI, Burrel M, Forner A, Sala M, et al (2007) Chemoembolization of hepatocellular carcinoma with drug eluting beads: Efficacy and doxorubicin pharmacokinetics. J Hepatology 46: 474–481. 10.1016/j.jhep.2006.10.020 17239480

[6] Thomas MB, Zhu AX (2005) Hepatocellularcarcinoma: the need forprogress. J ClinOncol 23: 2892–9.

[7] Llovet JM, Real MI, Montana X, Planas R, Coll S, et al (2002) Barcelona Liver Cancer Group: Arterial embolisation or chemoembolisation versus symptomatic treatment in patients with unresectable hepatocellular carcinoma: a randomised controlled trial. Lancet 359: 1734–1739. 12049862 10.1016/S0140-6736(02)08649-X

[8] Okada S, Okazaki N, Nose H, Shimada Y, Yoshimori M, Aoki K (1993) A phase 2 study of cisplatin in patients with hepatocellular carcinoma. Oncology 50 1: 2–26. 7678453 10.1159/000227142

[9] Falkson G, Moertel CG, Lavin P, Pretorius FJ, Carbone PP (1978) Chemotherapy studies in primary liver cancer: a prospective randomized clinical trial. Cancer 42 5: 2149–2156. 214218 10.1002/1097-0142(197811)42:5<2149::aid-cncr2820420510>3.0.co;2-5

[10] Lin HL, Liu TY, Chau GY, Lui WY, Chi CW (2000) Comparison of 2methoxyestradiol-induced, docetaxel-induced, and paclitaxel-induced apoptosis in hepatoma cells and its correlation with reactive oxygen species. Cancer 89 5: 983–994. 10964328

[11] Hamad I, Moghimi SM (2008) Critical issues in site-specific targeting of solid tumours: the carrier, the tumour barriers and the bioavailable drug. ExpertOpin Drug Deliv 5: 205–219. 10.1517/17425247.5.2.205 18248319

[12] Lo CM, Ngan H, Tso WK, Liu CL, Lam CM, et al (2002) Randomized controlled trial of transarteriallipiodol chemoembolization for unresectable hepatocellular carcinoma. Hepatology 35: 1164–1171. 11981766 10.1053/jhep.2002.33156

[13] Xu Z, Chen L, Gu W, Gao Y, Lin L, et al (2009) The performance of docetaxel-loaded solid lipid nanoparticles targeted to hepatocellular carcinoma. Biomaterials 30: 226–32. 18851881 10.1016/j.biomaterials.2008.09.014

[14] Bae S, Ma K, Kim TH, Lee ES, Oh KT, et al (2011) Doxorubicin-loaded human serum albumin nanoparticles surface-modified with TNF-related apoptosis-inducing ligand and transferrin for targeting multiple tumor types. Biomaterials 33: 1536–1546. 22118776 10.1016/j.biomaterials.2011.10.050

[15] Holmström P, Gåfvels M, Eriksson LC, Dzikaite V, Hultcrantz R, et al (2006) Expression of iron regulatory genes in a rat model of hepatocellular carcinoma. Liver Int 26: 976–85. 16953838 10.1111/j.1478-3231.2006.01316.x

[16] Tseng HH, Chang JG, Hwang YH, Yeh KT, Chen YL, et al (2009) Expression of hepcidin and other iron-regulatory genes in human hepatocellular carcinoma and its clinical implications. J Cancer Res Clin Oncol 135: 1413–20. 19387685 10.1007/s00432-009-0585-5 PMC12160254

[17] Krishna ADS, Mandraju RK, Kishore G, Kondapi AK (2009) An Efficient Targeted Drug Delivery through Apotransferrin Loaded Nanoparticles. PLoS ONE 4 10: e7240. 19806207 10.1371/journal.pone.0007240 PMC2752169

[18] Morgan EH, Smith GD, Peters TJ (1986) Uptake and subcellular processing of 59Fe-125I-labelled transferrin by rat liver. Biochem J 237: 163–173. 3800875 10.1042/bj2370163 PMC1146961

[19] Cao H, Krueger EW, McNiven MA (2011) Hepatocytes internalize trophic receptors at large endocytic “Hot Spots”. Hepatology 54: 1819–29. 21793030 10.1002/hep.24572 PMC3205295

[20] Fleming MD, Romano MA, Su MA, Garrick LM, Garrick MD, et al (1998) Nramp2 is mutated in the anemic Belgrade (b) rat: evidence of a role for Nramp2 in endosomal iron transport. ProcNatlAcadSci U S A 95: 1148–53. 10.1073/pnas.95.3.1148 PMC18702 9448300

[21] Zhao N, Gao J, Enns CA, Knutson MD (2010) ZRT/IRT-like protein 14 (ZIP14) promotes the cellular assimilation of iron through transferrin. J BiolChem 285: 32141–50. 10.1074/jbc.M110.143248 PMC2952215 20682781

[22] Cohn EJ, Strong LE, Hughes WL, Mulford DJ, Ashworth JN, et al (1964) Preparation and Properties of Serum and Plasma Proteins. IV. A System for the Separation into Fractions of the Protein and Lipoprotein Components of Biological Tissues and Fluids. J Am Chem Soc 68: 459–475. 10.1021/ja01207a034 21015743

[23] Sharma AK, Paramasivam M, Srinivasan A, Yadav MP, Singh TP (1999) Three-dimensional structure of mare diferriclactoferrin at 2.6Aresolution. J. Mol. Biol 289: 303–317. 10.1006/jmbi.1999.2767 10366507

[24] Zhang J, Guang X, Chen Y, Li Y, Liu CS (2007) Self-assembled nanoparticles based on hydrophobically modified chitosan as carriers for doxorubicin. Nanomedicine 3: 258–265. 17962086 10.1016/j.nano.2007.08.002

[25] Fiume L, Bolondi L, Busi C, Chieco P, Kratz F, et al (2005) Doxorubicincoupled to lactosaminatedalbumininhibits the growth of hepatocellularcarcinomasinduced in rats by diethylnitrosamine. J Hepatol 43: 645–52. 16023760 10.1016/j.jhep.2005.02.045

[26] Adams M, Buehner M, Chandrasekhar K, Ford G, Hackert M, et al (1973) Structure-Function Relationships in Lactate Dehydrogenase ,. ProcNatlAcadSci U S A 70: 1968. 10.1073/pnas.70.7.1968 PMC433644 4146647

[27] Ursini E, Maiorino M, Gregoin C (1985) Theselenoenzyme phospholipid hydroperoxide glutathione peroxidase. Biochem Biophys Acta 839: 62–70. 3978121 10.1016/0304-4165(85)90182-5

[28] Borbath I, Leclercq IA, Sempoux C, Abarca-Quinones J, Desaeger C, et al (2010) Efficacy of lanreotide in preventing the occurrence of chemically induced hepatocellular carcinoma in rats. ChemBiol Interact 183: 238–48. 10.1016/j.cbi.2009.10.011 19874807

[29] Wong HL, Bendayan R, Rauth AM, Wu XY (2006) Simultaneous delivery of doxorubicin and GG918 (Elacridar) by new polymer-lipid hybrid nanoparticles (PLN) for enhanced treatment of multidrug-resistant breast cancer. J Control Release 116: 275–284. 17097178 10.1016/j.jconrel.2006.09.007

[30] Lina R, Nga LS, Wang C (2005) In vitro study of anticancer drug doxorubicin in PLGA-based microparticles. Biomaterials 26: 4476–81. 15701377 10.1016/j.biomaterials.2004.11.014

[31] Gabizon A (2001) Pegylated liposomal doxorubicin: Metamorphosis of anold drug into a new form of chemotherapy. Cancer investigation 19: 424–36. 11405181 10.1081/cnv-100103136

[32] Gabizon A, Catane R, Uziely B, Kaufman B, Safra T (1994) Prolongedcirculation time and enhanced accumulation in malignant exudatesof doxorubicin encapsulated in polyethylene-glycol coated liposomes. Cancer Res 54: 98–992. 8313389

[33] SheelaA, AbrahamDN,WaterhouseLD,Mayer PRC, Thomas DM, et al..(2003) Liposomal formulation of doxorubicin. In D. Nejat (ed.),Methods in enzymology, vol. 391. Academic press 71–97. 10.1016/S0076-6879(05)91004-5 15721375

[34] Ahmed F, Pakunlu RI, Brannan A, Bates FS, Minko T, et al (2006) Biodegradable polymersomes loaded with both paclitaxel and doxorubicin permeate and shrink tumors, inducing apoptosis in proportion to accumulated drug. J Contr Release 116: 150–8. 10.1016/j.jconrel.2006.07.012 16942814

[35] Xu JP, Ji J, Chen W, Shen J (2005) Novel biomimetic polymersomes as polymer therapeutics for drug delivery. J Contr Release 107: 502–12. 10.1016/j.jconrel.2005.06.013 16154659

[36] Pang Z, Lu W, Gao H, Hu K, Chen J, et al (2008) Preparation and brain delivery property of biodegradable polymersomes conjugated with OX26. J Contr Release 128: 120–7. 10.1016/j.jconrel.2008.03.007 18436327

[37] Upadhyay KK, Bhat AN, Mishra AK, Chutani K, Dwarakanath BS, et al (2010) The intracellular drug delivery and antitumor activity of doxorubicin loaded poly(γ-benzyl L-glutamate)-bhyaluronanpolymersomes. Biomaterials 10: 2882–92. 10.1016/j.biomaterials.2009.12.043 20053435

[38] Kataoka K, Matsumoto T, Yokoyama M, Okano T, Sakurai Y, et al (2000) Doxorubicin-loaded poly (ethylene glycol)-poly([beta]-benzyl–aspartate) copolymer micelles: their pharmaceutical characteristics and biological significance. J Contr Release 64: 143–53. 10.1016/s0168-3659(99)00133-9 10640653

[39] Yooand HS, Park TG (2001) Biodegradable polymeric micelles composed of doxorubicin conjugated PLGA-PEG block copolymer. J Contr Release 70: 63–70. 10.1016/s0168-3659(00)00340-0 11166408

[40] Shuai X, Ai H, Nasongkla N, Kim S, Gao J (2004) Micellar carriers based on block copolymers of poly([var epsilon]-caprolactone) and poly(ethylene glycol) for doxorubicin delivery. J Contr Release 98: 415–26. 10.1016/j.jconrel.2004.06.003 15312997

[41] Fundarò A, Cavalli R, Bargoni A, Vighetto D, Zara GP, et al (2000) Non-stealth and stealth solid lipid nanoparticles (SLN) carrying doxorubicin: pharmacokinetics and tissue distribution after i.v. administration to rats. Pharmacol Res 42: 337–43. 10987994 10.1006/phrs.2000.0695

[42] Zara GP, Cavalli R, Bargoni A, Fundarò A, Vighetto D, et al (2002) Intravenous administration to rabbits of non-stealth and stealth doxorubicin-loaded solid lipid nanoparticles at increasing concentrations of stealth agent: pharmacokinetics and distribution of doxorubicin in brain and other tissues. J Drug Target 10: 327–35. 12164381 10.1080/10611860290031868

[43] Al-Abd AM, Hong KY, Song SC, Kuh HJ (2010) Pharmacokinetics of doxorubicin after intratumoral injection using a thermosensitive hydrogel in tumor-bearing mice. J Control Release 142: 101–7. 19819274 10.1016/j.jconrel.2009.10.003

[44] Schneider Y, Abarca J, Aboud-Pirak E, Baurain R, Ceulemans F, et al.. (1984) Drug targeting in human cancerchemotherapy. In: Gregoriadis G, Poste G, Senior J, Trouet A, editors. Receptor-mediated targeting of drugs. NATO ASI series A: life sciences 1–25.

[45] Fiume L, Bolondi L, Busi C, Chieco P, Kratz F, et al (2005) Doxorubicin coupled to lactosaminated albumin inhibits the growth of hepatocellular carcinomas induced in rats by diethylnitrosamine. J Hepatology 43: 645–652. 10.1016/j.jhep.2005.02.045 16023760

[46] Yokoyama M, Fukushima S, Uehara R, Okamoto K, Kataoka K, et al (1998) Characterization of physical entrapment and chemical conjugation of adriamycin in polymeric micelles and their design for in vivo delivery to a solid tumor. J Control Release 50: 79–92. 9685875 10.1016/s0168-3659(97)00115-6

[47] Chiannilkulchai N, Driouich Z, Benoit JP, Parodi AL, Couvreur P (1989) Doxorubicin-loaded nanoparticles: increased efficiency in murine hepatic metastases. Sel Cancer Ther 5: 1–11. 2756244 10.1089/sct.1989.5.1

[48] Chiannilkulchai N, Ammoury N, Caillou B, Devissaguet JP, Couvreur P (1990) Hepatic tissue distribution of doxorubicin-loaded nanoparticles after i.v. administration in reticulosarcoma M 5076 metastasis-bearing mice. Cancer Chemother Pharmaco l26: 122–126. 10.1007/BF02897257 2189589

[49] Verdun C, Brasseur F, Vranckx H, Couvreur P, Roland M (1990) Tissue distribution of doxorubicin associated with polyisohexylcyanoacrylate nanoparticles. Cancer Chemother Pharmacol 26: 13–18. 2322986 10.1007/BF02940287

[50] Alhareth K, Vauthier C, Bourasset F, Gueutin C, Ponchel G, et al (2012) Conformation of surface-decorating dextran chains affects the pharmacokinetics and biodistribution of doxorubicin-loaded nanoparticles. Eur J Pharm Biopharm 81 2: 453–7. 22465096 10.1016/j.ejpb.2012.03.009

[51] Zara GP, Cavalli R, Fundarò A, Bargoni A, Caputo O, et al (1999) Pharmacokinetics of doxorubicin incorporated in solid lipid nanospheres (SLN). Pharmacol Res 40 3: 281–6. 10479474 10.1006/phrs.1999.0509

[52] Ayen WY, Kumar N (2012) In Vivo Evaluation of Doxorubicin-Loaded (PEG)(3)-PLA Nanopolymersomes (PolyDoxSome) Using DMBA-Induced Mammary Carcinoma Rat Model and Comparison with Marketed LipoDox™. Pharm Res PMID 22669705. 10.1007/s11095-012-0783-8 22669705

[53] Li X, Ding L, Xu Y, Wang Y, Ping Q (2009) Targeted delivery of doxorubicin using stealth liposomes modified with transferrin. Int J Pharm 373 1–2: 116–23. 19429296 10.1016/j.ijpharm.2009.01.023

