# Supplementary material for: Efficacy, Safety and Anticancer Activity of Protein Nanoparticle-Based Delivery of Doxorubicin through Intravenous Administration in Rats
Source: PLoS One. 2012 Dec 21;7(12):e51960. doi: 10.1371/journal.pone.0051960 (PMC3528733; doi:10.1371/journal.pone.0051960)
Supplement: Table S1 — The encapsulation efficiency of nanoformulations was measured in nanopartciles prepared with 10 mg of protein and 10 mg of drug. When drug and protein was estimated in nanopartciles, the results showed that Apotransferrin and Lactoferrin encapsulated 66% and 79% respectively. (DOCX) [file pone.0051960.s001.docx]

**Supporting data table-1**

**Encapsulation efficiency**

|  | **The composition in feed (mg)** | | **The composition of drug loaded nanoparticles (mg)** | |
| --- | --- | --- | --- | --- |
|  | Protein | Doxorubicin | Protein | Doxorubicin |
| Apotransferrin | 10/ 100 µl | 2.5/ 100 µl | 8.7 ± 0.6 | 2.3± 0.1 |
|  | 10/ 100 µl | 5/ 200 µl | 8.7 ± 0.6 | 4.6 ± 0.2 |
|  | 10/ 100 µl | 7.5/ 300 µl | 8.7 ± 0.6 | 6.1 ± 0.3 |
|  | 10/ 100 µl | 10/ 400 µl | 8.7 ± 0.6 | 6.6 ± 0.3 |
| Lactoferrin | 10/ 100 µl | 2.5/ 100 µl | 8.9 ± 0.5 | 2.4 ± 0.3 |
|  | 10/ 100 µl | 5/ 200 µl | 8.9 ± 0.5 | 4.7 ± 0.3 |
|  | 10/ 100 µl | 7.5/ 300 µl | 8.9 ± 0.5 | 7.2 ± 0.2 |
|  | 10/ 100 µl | 10/ 400 µl | 8.9 ± 0.5 | 7.9 ± 0.2 |
