# Supplementary material for: Efficacy, Safety and Anticancer Activity of Protein Nanoparticle-Based Delivery of Doxorubicin through Intravenous Administration in Rats
Source: PLoS One. 2012 Dec 21;7(12):e51960. doi: 10.1371/journal.pone.0051960 (PMC3528733; doi:10.1371/journal.pone.0051960)
Supplement: Table S2 — The stability of nanoparticles was monitored at 4°C by incubation over 90 days. The results shows that both the formulations exhibited 2.5–5% drug loss when incubation period was between 24 hr to 90 days. (DOCX) [file pone.0051960.s002.docx]

Supporting data table 2S

Stability of nanoformulations

|  | Incubated at 4^o^C temp | Doxorubicin encapsulated  mg | Drug released  mg | Drug lost  mg |
| --- | --- | --- | --- | --- |
| Apodoxonano | 24 hr | 6.6 ± 0.3 | 6.435 | 0.165 |
|  | 30 days | 6.6 ± 0.3 | 6.402 | 0.198 |
|  | 60 days | 6.6 ± 0.3 | 6.336 | 0.264 |
|  | 90 days | 6.6 ± 0.3 | 6.27 | 0.33 |
| Lactodoxonano | 24 hr | 7.9 ± 0.2 | 7.7025 | 0.1975 |
|  | 30 days | 7.9 ± 0.2 | 7.663 | 0.237 |
|  | 60 days | 7.9 ± 0.2 | 7.584 | 0.316 |
|  | 90 days | 7.9 ± 0.2 | 7.505 | 0.395 |
